# Multiple-endpoint in vitro carcinogenicity test in human cell line TK6 distinguishes carcinogens from non-carcinogens and highlights mechanisms of action

**DOI:** 10.1007/s00204-020-02902-3

**Published:** 2020-09-10

**Authors:** Katherine E. Chapman, Eleanor C. Wilde, Fiona M. Chapman, Jatin R. Verma, Ume-Kulsoom Shah, Leanne M. Stannard, Anna L. Seager, James A. Tonkin, M. Rowan Brown, Ann T. Doherty, George E. Johnson, Shareen H. Doak, Gareth J. S. Jenkins

**Affiliations:** 1 In Vitro Toxicology Group, Institute of Life Science 1, Swansea University Medical School, Swansea University, Singleton Campus, Swansea, SA2 8PP UK; 2 College of Engineering, Swansea University, Bay Campus, Swansea, SA1 8EN UK; 3 Discovery Safety, AstraZeneca, DSM, Darwin Building, Cambridge Science Park, Milton Road, Cambridge, CB4 0WG UK

**Keywords:** In vitro, Carcinogenicity testing, Genotoxicity, Multiple endpoints

## Abstract

Current in vitro genotoxicity tests can produce misleading positive results, indicating an inability to effectively predict a compound’s subsequent carcinogenic potential in vivo. Such oversensitivity can incur unnecessary in vivo tests to further investigate positive in vitro results, supporting the need to improve in vitro tests to better inform risk assessment. It is increasingly acknowledged that more informative in vitro tests using multiple endpoints may support the correct identification of carcinogenic potential. The present study, therefore, employed a holistic, multiple-endpoint approach using low doses of selected carcinogens and non-carcinogens (0.001–770 µM) to assess whether these chemicals caused perturbations in molecular and cellular endpoints relating to the Hallmarks of Cancer. Endpoints included micronucleus induction, alterations in gene expression, cell cycle dynamics, cell morphology and bioenergetics in the human lymphoblastoid cell line TK6. Carcinogens ochratoxin A and oestradiol produced greater Integrated Signature of Carcinogenicity scores for the combined endpoints than the “misleading” in vitro positive compounds, quercetin, 2,4-dichlorophenol and quinacrine dihydrochloride and toxic non-carcinogens, caffeine, cycloheximide and phenformin HCl. This study provides compelling evidence that carcinogens can successfully be distinguished from non-carcinogens using a holistic in vitro test system. Avoidance of misleading in vitro outcomes could lead to the reduction and replacement of animals in carcinogenicity testing.

**Electronic supplementary material:**

The online version of this article (10.1007/s00204-020-02902-3) contains supplementary material, which is available to authorized users.

## Introduction

Thousands of new chemical entities (NCEs) are generated each year, and all require initial safety testing to predict their human health implications. Exposure to certain agents can increase human cancer risk, due to genotoxicity or other mechanisms of carcinogenesis. Carcinogenicity assessment of NCEs is, therefore, necessary prior to chemical advancement within the pharmaceutical, food, agriculture, and general manufacturing industries.

Chemical safety assessment generally follows a tiered route, where initial in vitro genotoxicity test results determine whether subsequent in vivo genotoxicity and carcinogenicity investigation is performed (Rovida et al. 2015). It is well accepted by regulators that in vitro tests demonstrate low specificity, failing to successfully distinguish carcinogens from non-carcinogens (Pfuhler et al. 2011; Rovida et al. 2015). There are, therefore, two classes of in vitro positives: true positives, which are in vitro positives that cause carcinogenesis in in vivo follow-up tests, and ‘misleading’ positives, which are positive in vitro yet negative when subsequently tested in vivo. Such misleading positives are, therefore, only identified when the results are not replicated in follow-up in vivo carcinogenicity testing (Fowler et al. 2012a; Kirkland et al. 2005a, b; Kirkland et al. 2007; Thybaud et al. 2007). Factors such as the choice of cell type and excessive toxicity from high doses can affect the frequency of misleading positive results from in vitro genotoxicity tests (Fowler et al. 2012a, b, 2014; Shah et al. 2016). The choice of treatment type, number of test concentrations and timescale in vitro will also impact on the outcome for certain endpoints at low doses (Chapman et al. 2015, 2020).

High misleading positive rates have important consequences, including hindering the development of many chemicals with beneficial applications, such as in products and treatments (Fowler et al. 2012a). A second serious consequence of such misleading positives is the required performance of unnecessary animal testing to further investigate positive in vitro results that are later determined to be artefactual (Pfuhler et al. 2009). Improving in vitro tests for carcinogenicity prediction is, therefore, imperative for alignment with the 3Rs principle (Burden et al. 2015) and, therefore, avoiding ethical issues and resources associated with in vivo testing. Furthermore, initiatives such as Toxicity Testing in the Twenty-First Century are recognising that the use of human cell-based in vitro testing may confer greater human relevance than animal-based tests (Adeleye et al. 2015; Council 2007).

The advantages of multi-endpoint in vitro approaches for accurate prediction of in vivo carcinogenicity are increasingly being recognised (Benigni 2014; Bourcier et al. 2015; Breheny et al. 2011; McKim and James 2010). The parallel assessment of multiple, holistic endpoints may enable a broad range of carcinogenic mechanisms to be monitored and link to adverse outcome pathways (AOPs) (Burden et al. 2015). Previously, we have demonstrated that integrated in vitro endpoints show promise in distinguishing genotoxic carcinogens from non-genotoxic carcinogens, with results also correlating well with in vivo data (Wilde et al. 2018).

While in vitro genotoxicity tests have been studied previously for their ability to identify carcinogens and non-carcinogens accurately (Kirkland et al. 2005a, b; Kirkland et al. 2006), holistic approaches have not been comprehensively validated. Indeed, using multiple genotoxicity test systems has been demonstrated to increase sensitivity (Kirkland et al. 2005a, b); this supports the use of multiple endpoints to provide more information on compounds’ biological effects. It is possible that the traditional genotoxicity endpoints alone have limited relevance for cancer prediction, and cancer-relevant endpoints should instead be pursued, given that the next test stage usually involves carcinogenicity assessment (Steiblen et al. 2020).

The objective of the present study, therefore, was to establish a more informative in vitro test that could increase confidence in in vitro genotoxicity data, and potentially be incorporated into current test batteries. This was achieved by evaluating, for the first time, whether our multi-endpoint in vitro carcinogenicity approach could correctly predict in vivo carcinogenicity outcomes for both carcinogens and non-carcinogens. The chemicals selected for study are summarised in Table 1. Based on the existing literature, two carcinogens, ochratoxin A (OTA) and 17-ß-oestradiol (oestradiol), were selected to further evaluate the test strategy for carcinogens of which the mechanism is not fully understood. As well as the carcinogens, two different types of ‘non-carcinogens’ were tested to validate our holistic approach; ‘misleading’ in vitro positives and toxic non-carcinogens. The three misleading in vitro positive compounds tested were quercetin, 2,4-dichlorophenol (2,4-DCP) and quinacrine dihydrochloride (QDH) (Kirkland et al. 2008). While quercetin has often been referred to as a misleading in vitro positive, there is also a TD_50_ value available suggesting that it is not necessarily a misleading positive (Table 2). The small number of studies that did produce positive in vivo carcinogenicity results with quercetin have, however, been heavily criticised due to study design (Pamukcu et al. 1980; Program 1992). The three non-carcinogens selected were caffeine, cycloheximide and phenformin HCl (Kirkland et al. 2016; Bryce et al. 2017). By comparing the overall outcomes for the carcinogens with non-carcinogens, we can determine the suitability of our test strategy for correctly identifying new chemicals with carcinogenic potential.

**Table 1:** A summary of the compounds used in the study

Group	Compound name	Source of exposure/application	Cellular mechanisms/carcinogenic potential (where applicable)
Carcinogen	Ochratoxin A (OTA)	Food contaminant (cereal, wine, coffee) (Heussner and Bingle 2015)	IARC Group II carcinogen. Genotoxic (nephro)carcinogen (Boorman 1989; Dai et al. 2003; El Adlouni et al. 2000; Pfohl-Leszkowicz and Castegnaro 2005)
	β-oestradiol	Steroid hormone (reproductive)	IARC Group I carcinogen. Evidence for genotoxic and non-genotoxic mechanisms of carcinogenesis (Bryce et al. 2017; Hernández et al. 2013)
Misleading in vitro positives	Quercetin	Most abundant flavonoid in the human diet (Casella et al. 2014)	TD_50_ value suggests carcinogenic potential (Table 2). Consistent misleading in vitro positive but full mechanism not known
	2,4-DCP	Herbicide used in agriculture (Munro et al. 1992)	Possibly superoxide radical generation by decreasing superoxide dismutase in vitro (Bukowska 2003; Garg et al. 2001)
	Quinacrine dihydrochloride (QDH)	Antimalarial drug, non-surgical female sterilisation (Clarke et al. 2001)	Evidence of in vitro DNA intercalation but is not carcinogenic (Clarke et al. 2001)
Toxic non-carcinogens	Cycloheximide	Antibiotic	Protein synthesis inhibitor that causes cytotoxicity (Youngblom et al. 1989)
	Caffeine	Stimulant	Mitochondria-dependent apoptosis, ROS inducer that causes cytotoxicity (Bryce et al. 2017)
	Phenformin HCl	Biguanide antidiabetic (Kirkland et al. 2016)	Non-carcinogen, negative in vivo (Kirkland et al. 2016)

**Table 2:** TD_50_ data (Gold database, Lhasa database) for rodent carcinogenicity (where applicable) for the test compounds

Chemical	TD_50_ (mg/kg/day)
OTA	0.136
Oestradiol	1.0
Quercetin	10.1
2,4-DCP	Negative
Caffeine	Negative
Cycloheximide	Negative/ND
Phenformin HCl	Negative/ND
QDH	Not available/ND

*ND* no data

## Materials and methods

### Chemicals

Test chemicals were purchased from Sigma-Aldrich and stored according to the manufacturer’s instructions. OTA, QDH and quercetin were dissolved/diluted in dimethyl sulfoxide (DMSO) (Fisher Scientific), whereas 2,4-DCP, caffeine, cycloheximide and phenformin HCl were dissolved/diluted in dH_2_O. Oestradiol was dissolved/diluted in ethanol. Final concentrations of test chemicals within cell cultures ranged from 0 to 770 µM, and these were selected based on toxicity, as outlined below. Safety precautions, such as PPE and suitable waste disposal, were taken to protect users from exposure to hazardous compounds.

### Cell culture

The human lymphoblastoid cell line, TK6 (ECACC), was cultured in RPMI 1640 Medium (Life Technologies) supplemented with 10% donor horse serum (BDGentest) and 2 mM l-glutamine (Life Technologies). The cells were maintained in culture between 1 × 10^5^ and 1 × 10^6^ cells/ml. For all studies, cells were seeded at a density of 1 × 10^5^ cells/ml and cultured for 24 h prior to treatment commencement (37 °C, 5% CO_2_).

### Selection of doses for study

Doses were selected based on initial relative population doubling (RPD) data to ensure that excessive toxicity (> 50% RPD) did not occur. Following this, MN datasets were generated based on the defined dose range, and from MN data, a selected number of doses were chosen for study with further endpoints. If the chemical was positive in the MN assay, the NOEL (no observed effect level), LOEL (lowest observed effect level) and the dose producing 50% reduction in RPD was tested. If the chemical tested negative in the MN assay, doses within the initial dose range tested were then taken forward. The multiple-dose approach allowed dose-dependent trends to be identified, as well as provide an indication of safe exposure levels.

### Cytokinesis-blocked micronucleus assay

Frequency of chromosome damage in the form of micronuclei was analysed using the cytokinesis-blocked micronucleus (CBMN) assay. The protocol for Metafer analysis was as published previously (Seager et al. 2014). Timepoints used were either 4 h treatment + 23 h recovery, or 23 h treatment + 23 h recovery, unless otherwise stated. Cytochalasin B (4.5 µg/ml) was added at the commencement of the recovery period and this ensured that cells divided following the treatment period to allow observation of micronuclei (Fenech et al. 2003). For all other endpoints, 0 h recovery time was allowed following treatment to maximise the window for observing treatment-specific cell and molecular effects. A total of 9000 binucleate cells were scored per treatment per replicate. Relative population doubling (RPD) (%) (Fellows et al. 2008; Lorge et al. 2008) was measured in parallel cultures in the absence of cytochalasin B, with < 50% reduction in RPD relative to the vehicle control aimed for, in line with OECD requirements.

### Protein isolation and immunoblotting

To investigate p53 and phospho-p53 expression following treatment with test chemicals, protein isolation and immunoblotting were performed. A previously published method was followed (Brusehafer et al. 2014).

### Gene expression analysis

A shortlist of genes for qRT-PCR analysis was generated via mRNA microarray chip technology (Illumina) to measure genome-wide transcriptome alterations, as detailed by Wilde et al. (2018). qRT-PCR was completed for cyclin-dependent kinase inhibitor 1A (*CDKN1A*), choline kinase alpha (*CHKA*) and serine/threonine protein kinase (*SGK1*). A previously published method was followed (Brusehafer, et al. 2014). Primer sequences are available in Wilde et al. (2018).

### Cell cycle analysis

Flow cytometry assessed nucleated cells in the cell cycle phases of G1, S and G2/M after 4 h or 23 h. Samples were harvested using the In Vitro MicroFlow Micronucleus Analysis Kit (Litron Laboratories), as per the manufacturer’s instructions. Samples were analysed using the BD Facs Aria Flow Cytometer (BD Biosciences), with FacsDiva software (BD Biosciences), as described in Verma et al. (2017). Appropriate gating was applied to segregate the cell populations within the respective cell cycle phases and a total of 36,000 events were analysed across three replicates for each treatment.

### Cell and nuclear morphology analysis

Cell and nuclear morphology analysis was performed using the INCell Analyzer 2000 followed by a MATLAB-based script to identify cells and nuclei from captured images. The full methodology was previously outlined by Wilde et al. (2018). For the toxic non-carcinogens, the CellProfiler 2.2.0 software was used to obtain equivalent data on cell and nuclear morphology.

### Bioenergetics studies

The Seahorse Bioanalyzer (Agilent) was used to measure bioenergetic flux in control and treated samples, to establish whether chemicals influenced mitochondrial activity. Seahorse analyses were performed as outlined by Wilde et al. (2018).

### ToxPi™ graphical user interface

**Fig. 1:**
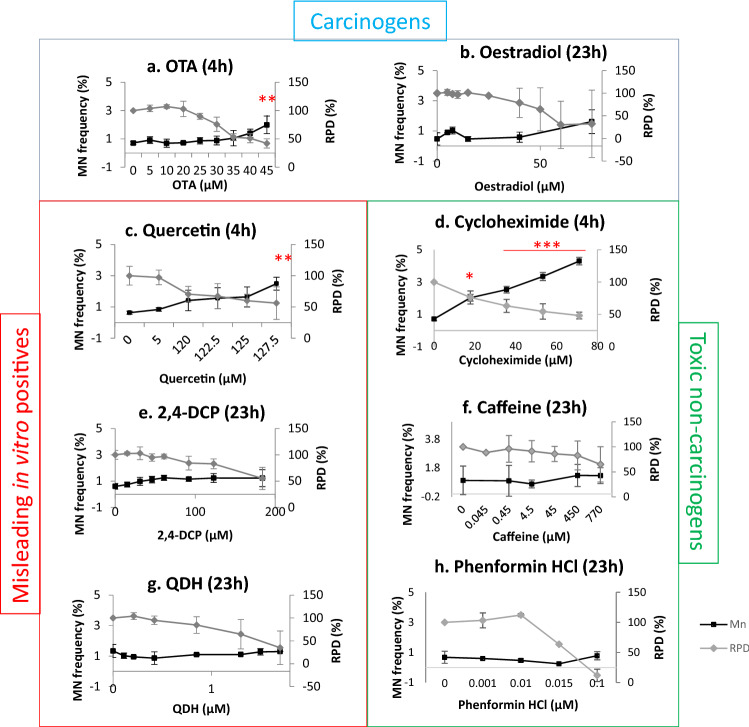
The cytokinesis-blocked micronucleus (CBMN) assay (4 h/23 h treatment + 23 h recovery) was used to assess whether test compounds induced genotoxicity. The percentage of binucleated cells containing micronuclei (MN, %) (black lines) and relative population doubling (RPD, %) (grey lines) are presented for the chemicals (*n* = 2, *n* = 3). Statistically significant results from the statistical analysis (Dunnett’s tests) are indicated by *p* values, where *p < 0.05, **p < 0.01, ***p < 0.001 (color figure online)

The Toxicological Prioritization Index (ToxPi™) graphical user interface (GUI) was used to generate overall profiles for the eight test chemicals (Reif et al. 2013). ‘Slices’ of the pie chart were weighted as necessary and the length of the radius was proportional to the magnitude of the change relative to the vehicle control. The concentration of chemical inducing an approximately 50% reduction in RPD relative to the vehicle control, or the highest concentration administered, was used to generate fold-change values relative to the control. The selection of the dose eliciting approximately 50% RPD was performed based on visual inspection of the original dose–response (Fig. 1). This dose was chosen with the objective of maximising the differentiation between carcinogens and non-carcinogens, given that the effect would be greatest at the highest concentration. The square root of all values was taken, and scores were scaled sufficiently to enable clear visualisation of all segments.

### Statistical analysis

Three biological replicates (except where indicated) were performed as independent experiments on separate days, with separate stock vials of cells/chemicals. Error bars represent standard deviation. Data were tested for normality (Shapiro–Wilk test) and homogeneity of variance (Levene’s statistic) and transformed where appropriate, prior to statistically significant changes being determined using a one-way ANOVA with appropriate post hoc tests depending on initial test outcomes. A mean-centering approach was used for the qRT-PCR data (Willems et al. 2008), prior to statistical analysis. Outcomes of *p* ≤ 0.05 for two-sided tests were deemed statistically significant. On all figures, statistically significant changes relative to the vehicle control were indicated using asterisks; *p ≤ 0.05; **p ≤ 0.01; ***p ≤ 0.001.

## Results

The present study applied an integrated test approach for carcinogenicity prediction within an in vitro system to observe for the first time whether carcinogens could be distinguished from non-carcinogens. Holistic endpoint analysis was investigated using eight test chemicals: carcinogens, OTA and oestradiol; misleading in vitro positives, 2,4-DCP, quercetin and QDH; non-carcinogens, caffeine, cycloheximide and phenformin HCl.

### Multiple test chemicals caused MN formation

The CBMN assay was used to generate genotoxicity dose–responses for all test chemicals (Fig. 1). Up to a 50% decrease in concurrent RPD was tolerated, to avoid secondary toxicity associated with higher concentrations. Chemicals were tested initially using a 4 h treatment with 23 h recovery; if the result after 4 h + 23 h was negative, the chemical exposure was then repeated for 23 h + 23 h. After 4 h, OTA produced statistically significant increases in MN frequency relative to the vehicle control. For OTA, the increase occurred at a concentration inducing an approximately 50% reduction in RPD (45 µM). Oestradiol produced a negative result after the 4 h and 23 h treatments, although was previously found to be positive in MCL-5 cells for an extended treatment period of 48 h + 23 h (Chapman 2018). To determine whether the 23 h result was a false negative, the longer exposure of 48 h + 23 h recovery was performed. Similar concentrations of oestradiol were used for 23 h and 48 h, given that the elongated exposure did not result in considerably elevated cytotoxicity. This resulted in a positive response for micronucleus induction for oestradiol at concentrations of 40 µM and higher (Online resource 1).

Quercetin showed genotoxic activity in this study after 4 h treatment at 127.5 µM only (50% RPD). Cycloheximide produced a statistically significant increase at all concentrations tested following 4 h exposure. While cycloheximide is toxic, it is not always considered to be genotoxic (Bryce et al. 2017); it was, therefore, decided that testing a 23 h exposure was necessary to confirm whether the 4 h result was a true positive (Online resource 1). A lower dose range was used for 23 h compared to 4 h, due to the elongated exposure period resulting in cytotoxicity at the higher doses tested. MN induction was greater after 4 h, where all concentrations caused significant MN induction and up to fivefold above vehicle control, compared to 23 h, where only one concentration was significant and the number of MN induced lower.

After 23 h, a reduced genotoxic response was noted with only one concentration of cycloheximide, 1.1 µM, being significant, whereas the higher concentration of 1.4 µM was negative. It was noted that the concentration window was particularly narrow, ranging between 0.7 and 1.4 µM. The remaining chemicals, 2,4-DCP, caffeine, phenformin HCl and QDH, did not significantly increase micronucleus frequency after either 4 h (data not shown) or 23 h treatments.

### OTA and quercetin increased both p53 and phospho-p53 expression

**Fig. 2:**
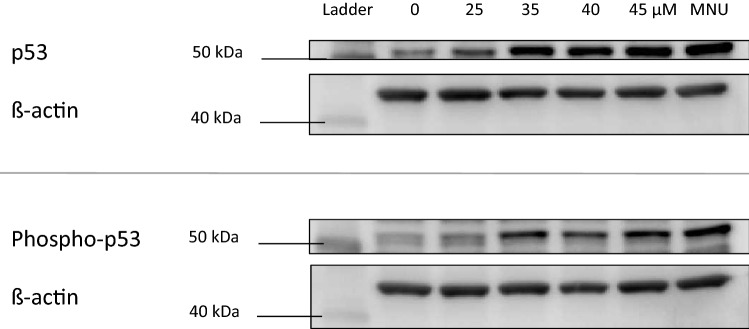
The change in expression of p53 and phospho-p53 was determined using western blotting. Representative blots are presented for OTA-treated cell cultures (4 h treatments), where *L* Ladder of protein standards and *N*-methyl-*N*-nitrosourea (MNU) were used as a positive control. The sizes of protein standard markers are labelled. Doses selected were based on the MN and RPD data generated previously (Fig. 1)

Western blotting was used to observe alterations in p53 and phospho-p53 (ser15) expression in response to treatment with the test chemicals, relative to the equivalent vehicle control (Fig. 2). The doses for western blotting and all subsequent endpoints were selected based on the LOEL and/or reduction in RPD (Fig. 1). A marked increase in p53 accumulation was noted for OTA at ≥ 35 µM after 4 h treatment for both p53 and phospho-p53 expression. The p53 induction was consistent with the positive MN data for OTA (Fig. 1), and indeed the LOEL for p53 and phospho-p53 increases was at a lower concentration than the LOEL for MN induction (45 µM). Oestradiol appeared to increase p53 and phospho-p53 expression at concentrations exceeding 25 µM, although this was not consistently observed across all replicates. This weaker response perhaps linked into the longer treatment required to induce MN (Online resource 1).

Of the remaining chemicals, quercetin significantly increased expression for concentrations ≥ 125 µM (Online resource 2). Like oestradiol, 2,4-DCP increased p53 expression following 184 µM exposure yet this was not observed for all replicates. QDH and the toxic non-carcinogens did not alter p53 or phospho-p53 expression.

### Carcinogens produced sizeable gene expression alterations

**Fig. 3:**
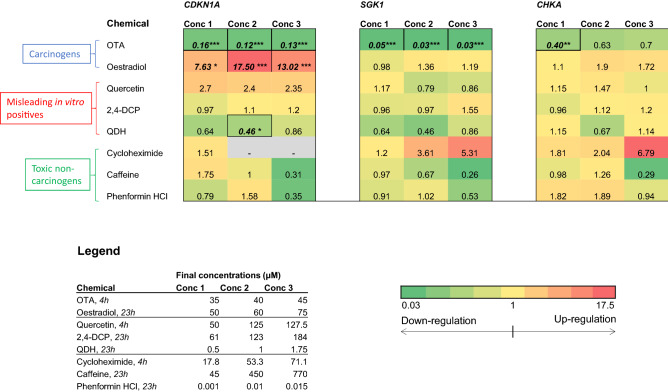
Heat maps summarizing the relative mRNA expression of the *CDKN1A*, *CHKA* and *SGK1* genes as determined by qRT-PCR (*n* = 3) for 4 h or 23 h exposure. Numbers indicate mean fold change relative to the appropriate vehicle control (equal to 1). Individual cells are coloured according to the magnitude of the fold change; shades of green represent a decrease relative to the vehicle control and shades of red represent an increase relative to the vehicle control. Significant results from the statistical analysis (Dunnett’s tests) are indicated by *p* values, where *p < 0.05, **p < 0.01, ***p < 0.001 (color figure online)

The transcription of three genes associated with cancer, *CDKN1A*, *SGK1* and *CHKA*, were measured using qRT-PCR. Fold change results were summarised using a heat map (Fig. 3). The carcinogens OTA and oestradiol significantly altered the expression of *CDKN1A* mRNA, which encodes p21. Oestradiol significantly increased *CDKN1A* mRNA expression at doses ≥ 50 µM, reaching a maximum increase of 17.5-fold at a concentration of 60 µM. This is consistent with the previously observed positive p53 response, indicative of a DNA damage response. In contrast to oestradiol, OTA appeared to suppress *CDKN1A* mRNA expression. OTA produced significant decreases in *CDKN1A* mRNA expression at all test concentrations, reaching a 7.7-fold decrease at the highest concentration of 45 µM. A decreasing trend was unexpected, due to OTA causing increased MN, p53 and phospho-p53 levels. The expression of the two remaining genes, *SGK1* and *CHKA*, was only altered by OTA. For *SGK1*, all OTA test concentrations produced a statistically significant decrease in expression, reaching a > 33.3-fold decrease at 45 µM. For *CHKA*, a single significant decrease of 35 µM was observed for mRNA expression. While toxic non-carcinogen cycloheximide substantially increased gene expression, the data were variable and not significant. Generally, chemicals that altered gene expression also induced micronuclei (Fig. 1).

The only other chemical to alter gene expression was QDH, which reduced expression of *CDKN1A* by twofold at a single test concentration (1 µM). This effect was not dose dependent, as expression did not change significantly at the higher QDH dose of 1.75 µM. QDH did not, however, alter the related endpoints of MN or p53. Other than QDH, none of the misleading in vitro positives and toxic non-carcinogens significantly altered gene expression for the genes tested.

### Cell cycle arrest was time-dependent for several test chemicals

**Fig. 4:**
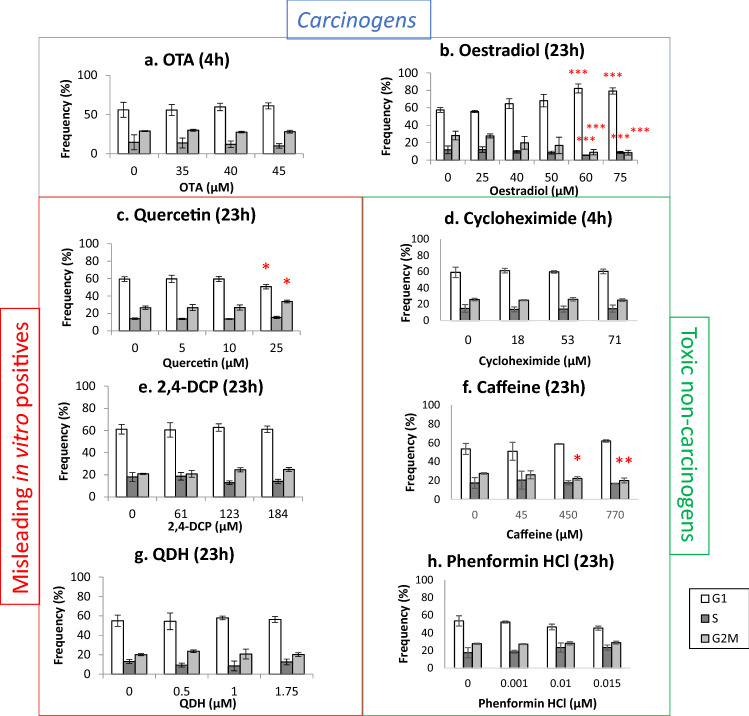
Cell cycle analysis was performed using flow cytometry for samples treated for 4 h or 23 h (*n* = 3) to determine whether chemicals altered cell cycle dynamics. Significant results from the statistical analysis (Dunnett’s tests) are indicated by *p* values, where *p < 0.05, **p < 0.01, ***p < 0.001

Flow cytometry was used to collect data on alterations in cell cycle dynamics following chemical treatments, to ascertain whether chemicals were capable of inducing cell cycle arrest (Fig. 4).

Oestradiol demonstrated a consistent dose-dependent trend towards G1 arrest, with an up to 21% significant increase in the percentage of cells in G1 phase occurring at 60 and 75 µM, accompanied by a significant decrease in the proportion of cells in both S and G2/M phases. This G1 cell cycle arrest is consistent with the large increases in *CDKN1A* mRNA caused by oestradiol (Fig. 3). OTA did not induce any significant cell cycle alterations after 4 h, although did cause a significant decrease in S phase at 4 h treatment with a recovery period (Online resource 3).

Following a 4 h exposure, quercetin did not induce significant cell cycle alterations, although a dose-dependent trend was observed (data not shown). To further investigate this apparent effect, and considering quercetin’s positive effects for some of the previously described endpoints, the exposure period was extended to 23 h, after which a statistically significant 7% increase in G2/M and a 8.7% decrease in G1 were observed at 25 µM, the highest test concentration for this time point. Lower chemical concentrations were used at 23 h relative to the initial 4 h studies for quercetin to avoid excessive toxicity. This outcome was consistent with the genotoxicity and p53 increases observed following quercetin exposure (Fig. 1, Online resource 2).

The other misleading in vitro positive compounds QDH and 2,4-DCP did not cause any significant cell cycle alterations following their respective 23 h exposures. The toxic non-carcinogens cycloheximide and phenformin HCl did not cause any statistically significant changes, although caffeine did induce a statistically significant decrease in cells in G2/M at 450 and 700 µM.

### Toxic non-carcinogens altered cell morphology

**Fig. 5:**
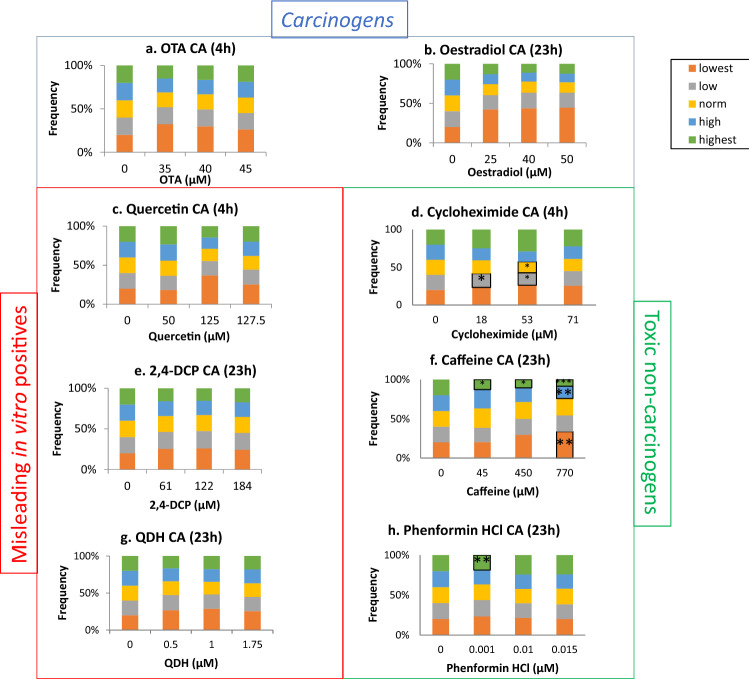
Cell area changes from data obtained via the INCell Analyzer 2000 followed by Matlab-based image analysis. The frequency of cells (%) in each quintile category is plotted. Significant results from the statistical analysis (Dunnett’s tests) are indicated by *p* values, where *p < 0.05, **p < 0.01, ***p < 0.001

**Fig. 6:**
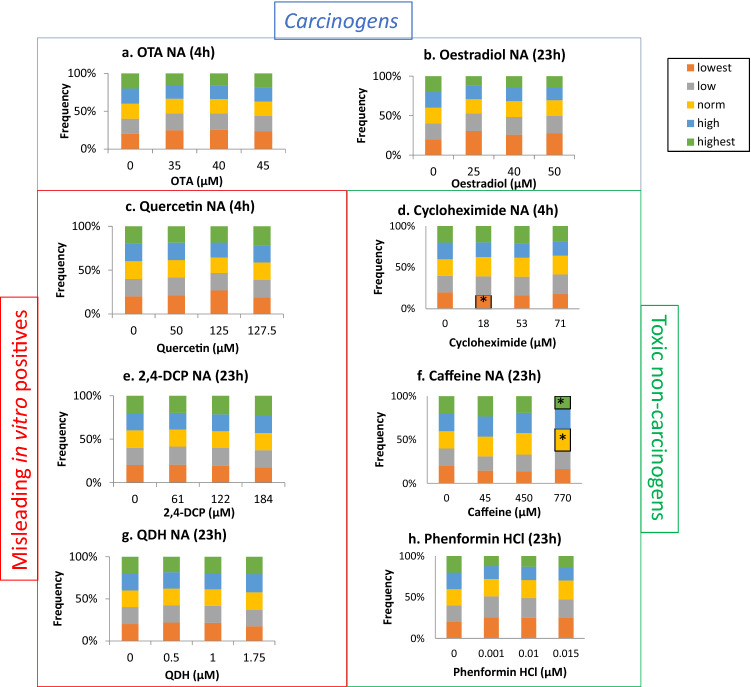
Nuclear area changes from data obtained via the INCell Analyzer 2000 followed by Matlab-based image analysis. The frequency of cells (%) in each quintile category is plotted (*n* = 3). Significant results from the statistical analysis (Dunnett’s tests) are indicated by *p* values, where *p < 0.05, **p < 0.01, ***p < 0.001

The INCell Analyzer 2000-based analyses were used to quantify cell and nuclear area alterations in response to test chemical exposure (Figs. 5, 6). Cells were treated for either 4 h or 23 h.

For cell and nuclear area, the tested carcinogens and misleading in vitro positive compounds did not produce any significant alterations. Oestradiol induced marked, but non-significant, changes in some cell area categories (Fig. 5). For example, there was a greater than twofold increase in the frequency of cells falling into the ‘lowest’ area category for all concentrations of oestradiol (≥ 25 µM) (*p* = 0.197 at 50 µM). Alongside this change, the frequencies of the largest area categories also decreased. This trend towards smaller cell area is consistent with oestradiol’s induction of cell cycle arrest at G1 (Fig. 4), which would produce smaller cells.

The only chemicals to produce significant changes were the toxic non-carcinogens. Caffeine produced statistically significant, dose-dependent decreases in the frequency of cells in the ‘highest’ size category, from 45 to 770 µM, and increased the frequency of cells in the ‘lowest’ category at 770 µM only (Fig. 5). These results suggest that caffeine reduced cell area. Cell area was also altered by cycloheximide and phenformin HCl, although statistically significant effects were observed only at lower concentrations and did not appear to be dose dependent.

For nuclear area, only cycloheximide and caffeine caused significant changes (Fig. 6). Cycloheximide reduced the proportion of the smallest, or ‘lowest’, nuclear area category for the lowest test concentration, 18 µM, only. Higher concentrations, however, did not cause statistically significant changes. Caffeine significantly changed the proportion of cells in the ‘normal’ and ‘highest’ categories following treatment after the highest treatment concentration of 770 µM, although there was not a clear result in terms of the direction of change of nuclear morphology. Overall, cell area appeared to be more sensitive for the detection of morphological changes caused by test chemicals than nuclear area.

### Mitochondrial activity was mostly unchanged

**Fig. 7:**
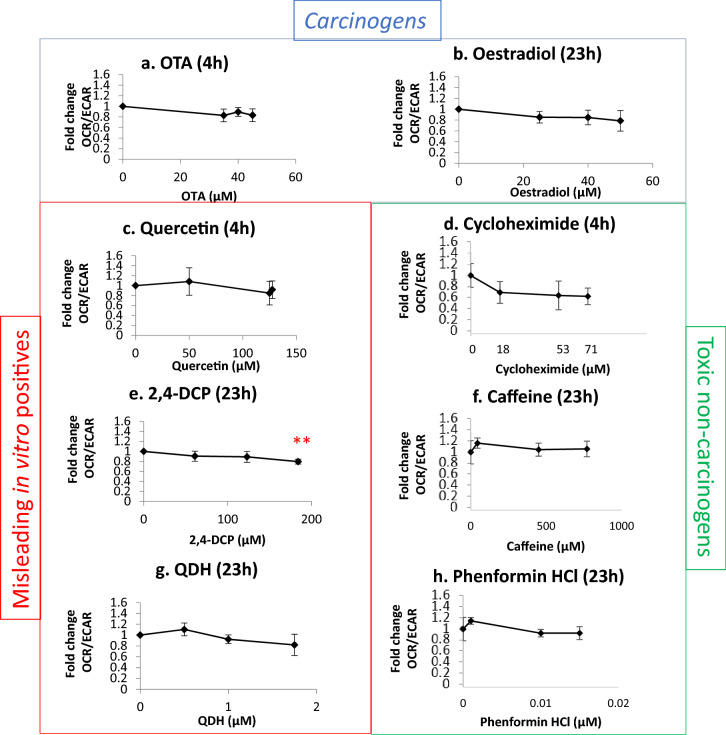
Bioenergetics analysis of control and treated cells using the Seahorse XF^e^24 Bioanalyzer (*n* ≥ 3) to establish whether chemicals induced a mitochondrial stress phenotype. The fold change for the ratio of oxygen consumption rate (OCR) and extracellular acidification rate (ECAR) is plotted against chemical concentration. Significant results from the statistical analysis (Dunnett’s tests) are indicated by *p* values, where *p < 0.05, **p < 0.01, ***p < 0.001

Bioenergetics analysis was completed using the Seahorse Bioanalyzer, to allow effects on mitochondrial respiration to be observed (Fig. 7). The two carcinogens did not cause any statistically significant changes for this endpoint; the sole chemical to produce a statistically significant alteration from the control was 2,4-DCP, producing a 20% reduction in mitochondrial activity after 23 h at the 50% RPD concentration of 184 µM. The data for several other chemical treatments indicated a similar decreasing trend in OCR/ECAR fold change with increasing test chemical concentration, although these alterations were not found to be statistically significant.

### ToxPi GUI indicated carcinogens’ greater potency

**Fig. 8:**
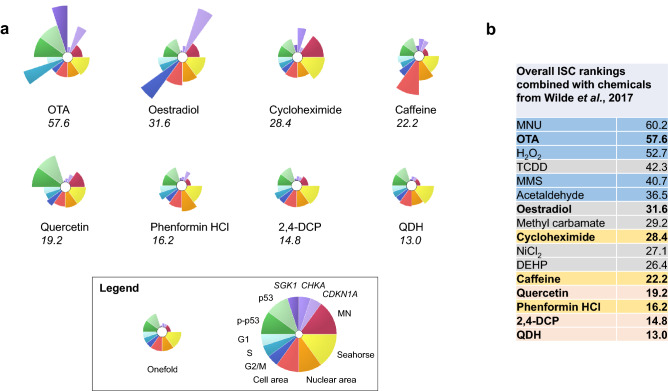
**a** Outputs from the Toxicological Prioritization Index (ToxPi) GUI summarising the fold changes for the endpoints at the 50% RPD-inducing concentration. Chemicals were ranked according to their Integrated Signature of Carcinogenicity scores, from highest to lowest. Fold changes were square-rooted and values < onefold were inverted to give values > onefold. **b** Table summarising ISCs for all chemicals tested using the multiple-endpoint method, including those from Wilde et al. (2018) (non-emboldened text). Chemicals in bold are from the current publication. Blue = genotoxic carcinogens; grey = non-genotoxic carcinogens; yellow = toxic non-carcinogens; pink = misleading in vitro positives (color figure online)

To visually summarise the results of the multiple-endpoint approach, the ToxPi GUI software was used to generate diagrammatical representations for all of the chemicals (Fig. 8a). Integrated Signature of Carcinogenicity (ISC) scores (Wilde et al. 2018) were also generated to quantitatively rank the chemicals based on their collective effects at the highest tested concentration that also did not exceed a 50% reduction in RPD (Fig. 1). The ISC scores were calculated based on the sum of the mean fold changes for the different endpoints, with endpoints weighted, as appropriate.

ISC scores demonstrated that carcinogens OTA and oestradiol were the highest ranking of the eight test chemicals, with OTA ranking first and oestradiol, second. The remaining test chemicals, cycloheximide, quercetin, caffeine, 2,4-DCP, phenformin HCl and QDH, ranked lower. These ISC scores were combined with ISC scores from the aforementioned publication by Wilde et al., where eight genotoxic and non-genotoxic carcinogens were studied (Fig. 8b). It was observed that carcinogens generally ranked higher than misleading in vitro positives and toxic non-carcinogens, with the exception of cycloheximide.

## Discussion

Improved in vitro genotoxicity tests are essential for the accurate prediction of the carcinogenic potential of chemicals in vivo and the avoidance of unnecessary animal tests. The present study aimed to evaluate a more sophisticated, multiple-endpoint in vitro approach for distinguishing between selected carcinogens, misleading in vitro positive compounds and toxic non-carcinogens, as compared to in vivo carcinogenicity outcomes where relevant.

### Outcomes for multiple endpoints in vitro indicated carcinogens’ mechanisms

The two tested carcinogens’ endpoint outcomes generally reflected their established mechanisms of carcinogenesis. First, the carcinogen OTA induced MN (Fig. 1), supporting a genotoxic mode of carcinogenesis for this agent (Table 1). OTA also increased p53 and phospho-p53 expression (Fig. 2) and altered gene expression (Fig. 3), which supported the MN results. It was noted that while MN frequency increased only at the highest concentration tested (45 µM), p53 and phospho-p53 were increased at doses below the lowest observed effect level for MN (Fig. 1). Equally, oestradiol elicited significant effects for non-MN endpoints after 23 h exposure, while only inducing MN after 48 h. This important observation demonstrated that other endpoints exhibited greater sensitivity than MN frequency and perhaps indicates the role of DNA repair at lower concentrations preventing eventual MN formation (Zaïr et al. 2011). The decreased expression of *CDKN1A* mRNA caused by OTA was unexpected, when it was considered that OTA increased the related endpoints of MN frequency and p53 expression. A similar phenomenon of p53 up-regulation accompanied by p21 inhibition has, however, been observed previously following OTA exposure and was hypothesised to be due to p53-independent inhibitory action of OTA on transcription (Golli Bennour et al. 2009). Indeed, decreased p21 is associated with a cancer phenotype (Gartel and Radhakrishnan 2005). OTA’s effects on the cell cycle were minimal, which supported the reduced p21 expression; such effects were also time-dependent, with 4 h treatment producing negative results (Fig. 4), whereas recovery led to reduced S phase frequency (Online resource 2).

Oestradiol, like OTA, was genotoxic yet was only positive after an elongated treatment period of 48 h (Fig. 1, Online resource 1). As for OTA, this indicates that exposure duration can be important for observing positive effects. While oestradiol was negative for MN after 23 h, this timepoint produced sizeable changes in other endpoints; for example, a large increase in *CDKN1A* mRNA accompanied by cell cycle arrest at G1 at concentrations ≥ 50 µM were observed. Oestradiol’s effects on the cell cycle have been documented previously (Yue et al. 2013). It could, however, be argued that G1 arrest would not be consistent with mismatch repair of associated replication errors (Yue et al. 2013), which is more likely to occur during G2 arrest (Hawn et al. 1995), implying alternative mechanisms were at work.

The two carcinogens did not alter cellular and nuclear morphology endpoints (Figs. 5, 6) and this contrasted with positive results for most carcinogens in Wilde et al. (2018). This might owe to the carcinogens selected here having different mechanisms and potency to those tested previously.

Overall, the carcinogens produced clear, dose-dependent and in some cases time-dependent responses for several endpoints, further supporting the use of integrated, multiple-endpoint testing approaches for recognising carcinogens.

### Misleading in vitro positive compounds and toxic non-carcinogens produced largely negative results

Following validation of the approach with carcinogens, it was essential to establish whether the non-carcinogens produced different results and did not erroneously test positive. To evaluate this, three misleading in vitro positive compounds and three toxic non-carcinogens were used.

Of the three misleading positives, quercetin elicited the greatest effect, inducing MN (Fig. 1), p53, phospho-p53 (Fig. 2) after 4 h treatments and G2/M arrest after 23 h (Fig. 4). The overall outcomes for quercetin suggested behaviour reminiscent of genotoxic carcinogens, such as OTA, as well as other compounds in this category (Wilde et al. 2018). Overall, these data imply that quercetin is fundamentally genotoxic under these conditions, which in vitro, has been attributed to auto-oxidation effects (Harwood et al. 2007). This provides cause to question whether classification of chemicals into discrete categories is an oversimplification (Wilde et al. 2018), and quercetin is a carcinogen, particularly as it has a TD_50_ value (Table 2).

In contrast, other misleading positive compounds, 2,4-DCP and QDH, produced a very limited number of positive results in a sporadic manner. QDH altered *CDKN1A* expression at 1 µM, yet not at the highest dose of 1.75 µM, suggesting that this effect was not dose dependent. Similarly, 2,4-DCP appeared to alter p53 levels, yet not phospho-p53, and the result was not consistent across experimental replicates. Inconsistency within 2,4-DCP test results has been reported previously (Fowler et al. 2012a). As 2,4-DCP did not increase MN frequency, the p53 result may have been an artefact of toxicity. The only endpoint that 2,4-DCP significantly altered was mitochondrial activity, being the only chemical to affect this endpoint (Fig. 7). A possible explanation is 2,4-DCP’s proposed involvement in in vitro superoxide radical generation (Garg et al. 2001). Interestingly, while QDH appeared almost inert, only inducing one positive outcome, it was also the most potent apart from phenformin HCl, eliciting a 50% reduction in RPD at lower molarity than most other chemicals (i.e., ≤ 1.75 µM).

Similar to the misleading positive chemicals, the toxic non-carcinogens cycloheximide, caffeine and phenformin HCl were also found to produce largely negative results. Cycloheximide had the largest effect, inducing MN at two different timepoints (Fig. 1, Online resource 1) and altering cell and nuclear area (Figs. 5, 6); however, these were not usually dose-dependent patterns, with sometimes only one, low test concentration producing a significant result. Despite being considered a non-carcinogen, previous studies have also demonstrated that cycloheximide can induce genotoxicity, including MN, in both in vitro and in vivo models (Sei-ichi et al. 1990; Bašić-Zaninović et al. 1991). Cycloheximide was shown to induce MN via both clastogenicity and aneugenicity (Basic-Zaninovic et al. 1987). Collectively, these results suggest that cycloheximide is a genotoxicant with carcinogenic potential; therefore, its classification as a non-carcinogen might not be accurate.

Caffeine produced positive results for three endpoints, with some dose-dependent effects. Caffeine reduced G2M frequency (Fig. 4) and reduced cell and possibly nuclear area (Figs. 5, 6), supporting cell cycle arrest in G1 phase. In support of such effects, caffeine has been demonstrated to induce *TP53*-independent G1 arrest in human cells (Qi et al. 2002), which supports this chemical’s negative western blot data. The positive results for caffeine could relate to toxic rather than carcinogen effects. Phenformin HCl, however, tested negative for all endpoints with the exception of a positive result for one quintile for cell area, although this was not dose dependent. Despite negative outcomes, it was the most potent agent given that the highest dose tested was 0.1 µM; this was a far lower molarity than that of other compounds.

Overall, these results indicate that while misleading in vitro positive chemicals and toxic non-carcinogens did produce a small number of positive results, these were generally not inter-supporting, not across multiple doses and of a smaller magnitude than for carcinogens. Compared to the carcinogens, therefore, overall effects for non-carcinogens were noticeably weaker within this test system. It is perhaps worth considering that a minimum ‘threshold’ number of concentrations or endpoints need to be significantly altered for a chemical to be classed as a ‘carcinogen’.

### ISC scores successfully distinguished carcinogens from non-carcinogens

ToxPi GUI analysis and ISC score generation enabled the observation of overall potency of the test chemicals based on all endpoints at the greatest test concentration (Fig. 8); the two carcinogens, OTA and oestradiol, produced the two highest ISC scores, with OTA ranking first (57.6) and oestradiol, second (31.6). This order also reflected the TD_50_ rank order in Table 2, with OTA’s lower TD_50_ indicating greater potency relative to oestradiol. The higher ranking of the genotoxic carcinogen, OTA, relative to non-genotoxic, or less potently genotoxic, carcinogen, oestradiol, was consistent with the results for these two carcinogen subtypes published previously (Wilde et al. 2018).

Other chemicals ranked below the two carcinogens in terms of ISC score (Fig. 8). While there was some overlap between toxic non-carcinogens and misleading positives, toxic non-carcinogens generally ranked higher with an average ISC of 22.3, compared to 15.7 for misleading positives. There did appear to be some overlap between non-carcinogen ISCs and non-genotoxic carcinogens ISCs published previously by Wilde et al. (2018); non-carcinogen cycloheximide’s ISC was 28.4, whereas non-genotoxic carcinogens NiCl_2_ and DEHP produced lower ISCs of 27.1 and 26.4, respectively. As previously mentioned, it is, however, possible that cycloheximide is inherently genotoxic (Fig. 1, Online resource 1) and so classification as a non-carcinogen might not fully reflect its biological activity. This is supported by the fact the second highest non-carcinogen ISC was 22.2, which was less than any carcinogen, genotoxic or non-genotoxic, tested. This suggests that the multiple-endpoint approach is less likely than single endpoint approaches to generate misleading positive results and may be superior for recognising carcinogenic potential and identifying such mechanisms of action. However, given that cycloheximide’s score exceeded that of two non-genotoxic carcinogens, further validation of the approach and perhaps the addition of other endpoints may be appropriate in future work.

The greater ISCs for carcinogens compared to non-carcinogens implied that carcinogens produced a greater biological effect for endpoints relating to the Hallmarks of Cancer (Hanahan and Weinberg 2011), supporting the use of such a test to identify chemicals’ carcinogenic potential. It was noted that multiple endpoints altered by carcinogens tended to be mechanistically inter-supporting. Changes were also often observed for at least two individual doses, occurring in a dose-dependent manner. Non-significant changes also contributed towards the overall ISC score, meaning that more subtle effects could assist in informing chemical risk assessment. These outcomes all support the use of an integrated and quantitative weight of evidence (WoE) approach for distinguishing between carcinogens and non-carcinogens in vitro, rather than isolated, single mode of action endpoints and tiered approaches (Rovida et al. 2015; Thybaud et al. 2007). Further study will assist in determining which endpoints are most powerful for distinguishing between carcinogens and non-carcinogens; the data from Wilde et al. (2018) and the present study suggest that MN, p53, *CDKN1A* and cell cycle data may be among the most powerful.

## Conclusions

In vitro genotoxicity tests remain rudimentary and often fail to successfully distinguish carcinogens from non-carcinogens. In the first study of its kind, we have provided compelling evidence for a human cell-based, multiple-endpoint in vitro carcinogenicity test distinguishing between carcinogens and non-carcinogens. This holistic approach also identifies mechanisms of carcinogenic action in vitro, while identifying results that are not dose dependent. With further validation, it is hoped that the ranking of chemicals based on their ISC scores may allow a minimum ‘cut-off’ score for carcinogens to be established. This could support the avoidance of misclassifying non-carcinogens as carcinogens via in vitro test results. Indeed, we have demonstrated that there is potential for applying holistic approaches to in vitro 3D cell culture models in future (Chapman et al. 2014; Shah et al. 2018). Overall, holistic approaches appear to be a valuable tool for identifying non-carcinogens at the in vitro stage, avoiding unnecessary in vivo testing.

## Electronic supplementary material

Below is the link to the electronic supplementary material.

Supplementary file1 (PPTX 138 kb)

## Data Availability

Will be made available.

## Code availability

Will be made available.
