# Supplementary material for: Multiple-endpoint in vitro carcinogenicity test in human cell line TK6 distinguishes carcinogens from non-carcinogens and highlights mechanisms of action
Source: Arch Toxicol. 2020 Sep 10;95(1):321–36. doi: 10.1007/s00204-020-02902-3 (PMC7811515; doi:10.1007/s00204-020-02902-3)

## Slide 1
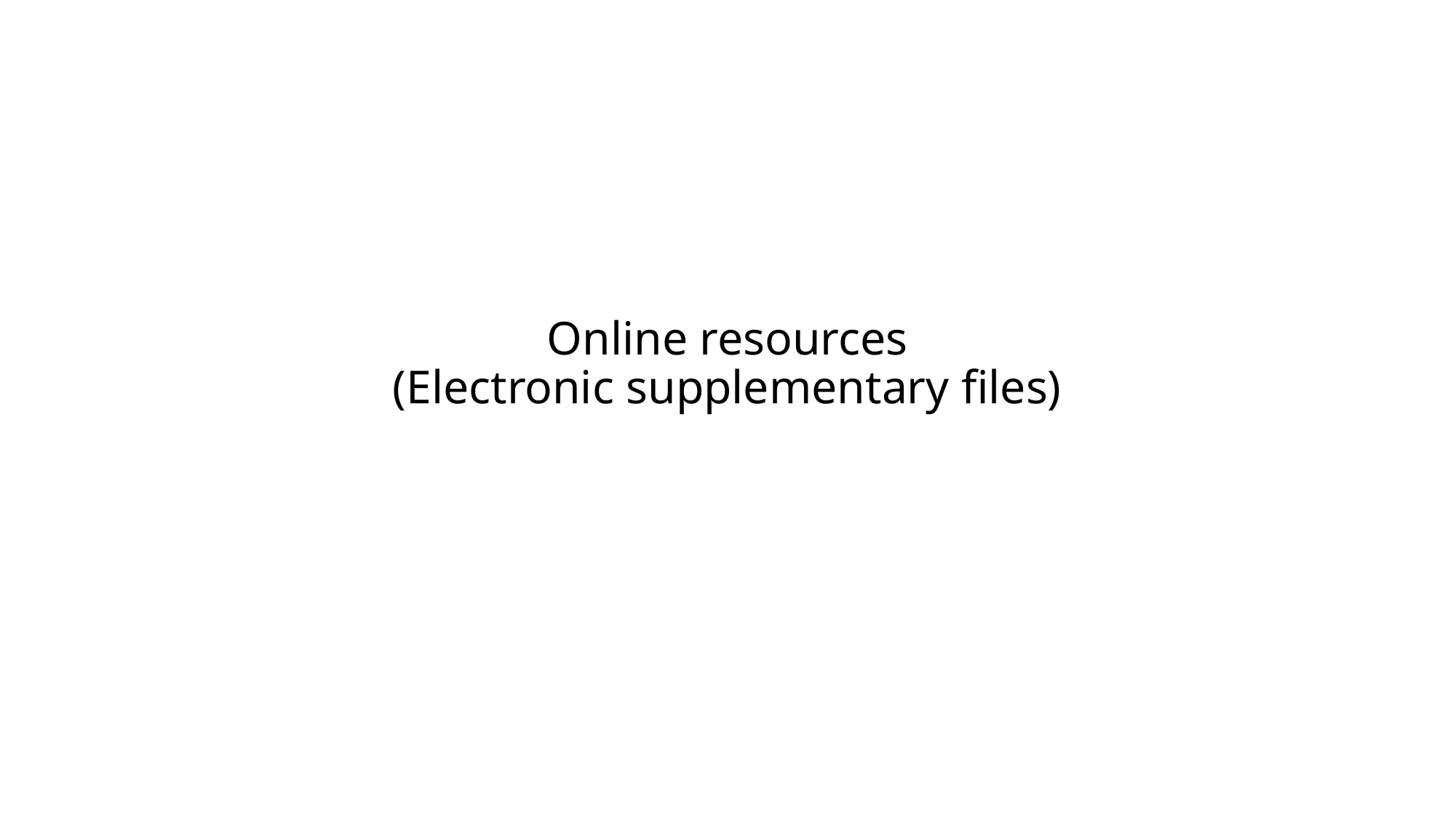

# Online resources (Electronic supplementary files)

## Slide 2
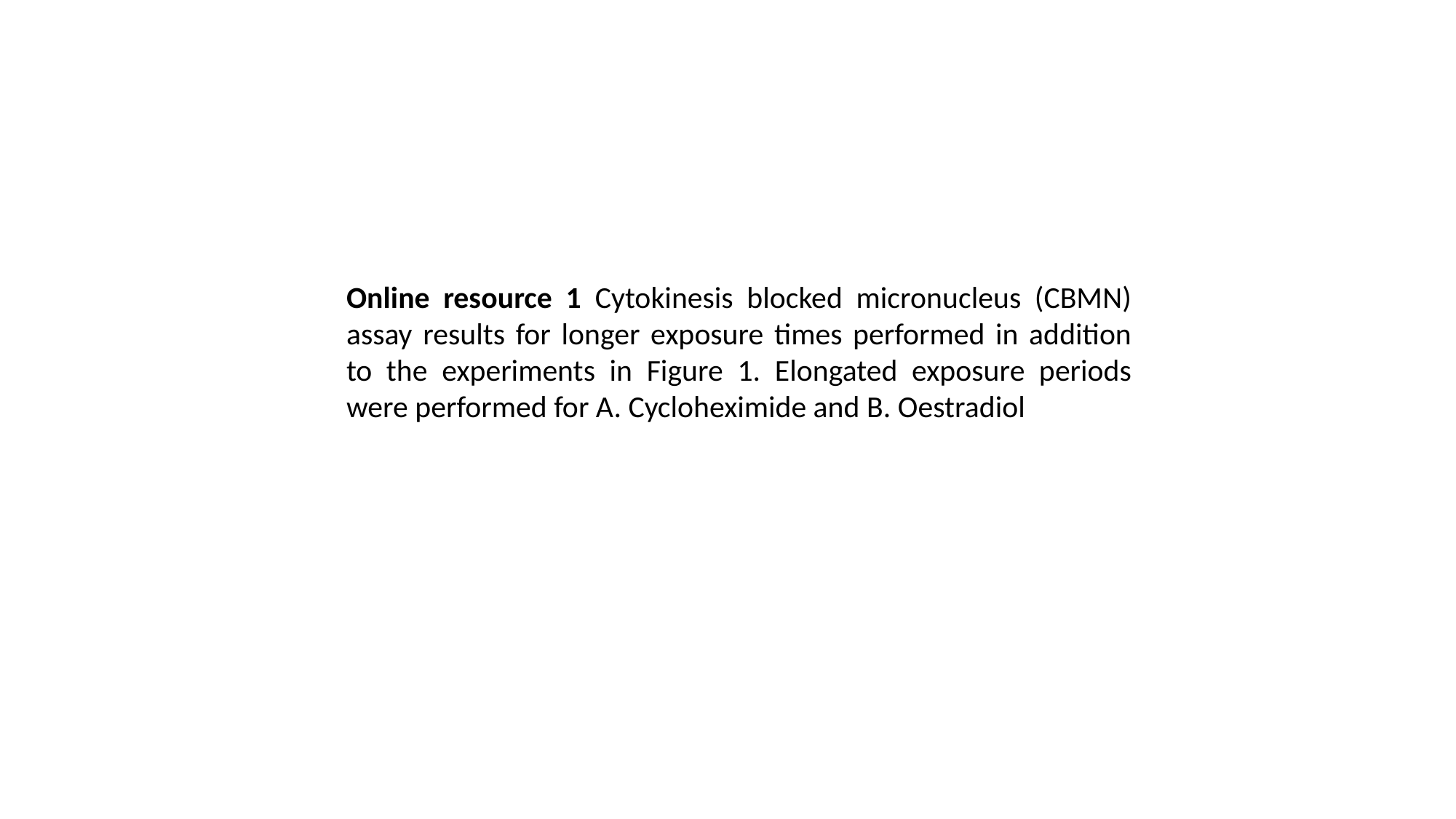

Online resource 1 Cytokinesis blocked micronucleus (CBMN) assay results for longer exposure times performed in addition to the experiments in Figure 1. Elongated exposure periods were performed for A. Cycloheximide and B. Oestradiol

## Slide 3
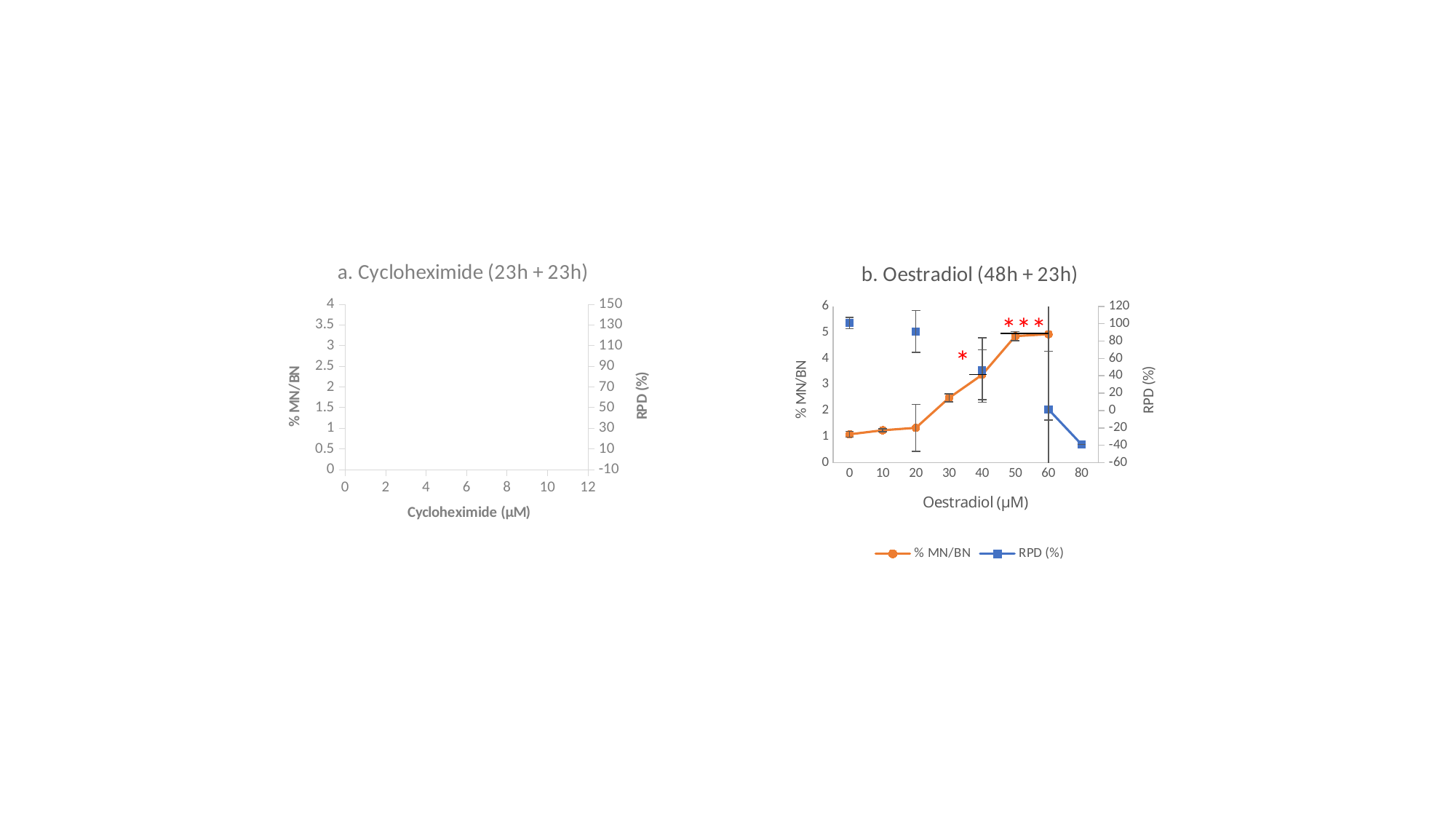

### Chart: a. Cycloheximide (23h + 23h)
| Category | | %RPD |
|---|---|---|
### Chart: b. Oestradiol (48h + 23h)
| Category | % MN/BN | RPD (%) |
|---|---|---|
| 0 | 1.0875 | 100.80487874919883 |
| 10 | 1.2425 | None |
| 20 | 1.3375 | 91.13386901826274 |
| 30 | 2.4875 | None |
| 40 | 3.375 | 46.64682341924622 |
| 50 | 4.8575 | None |
| 60 | 4.93 | 1.386756138460676 |
| 80 | None | -39.087968160430755 |***

## Slide 4
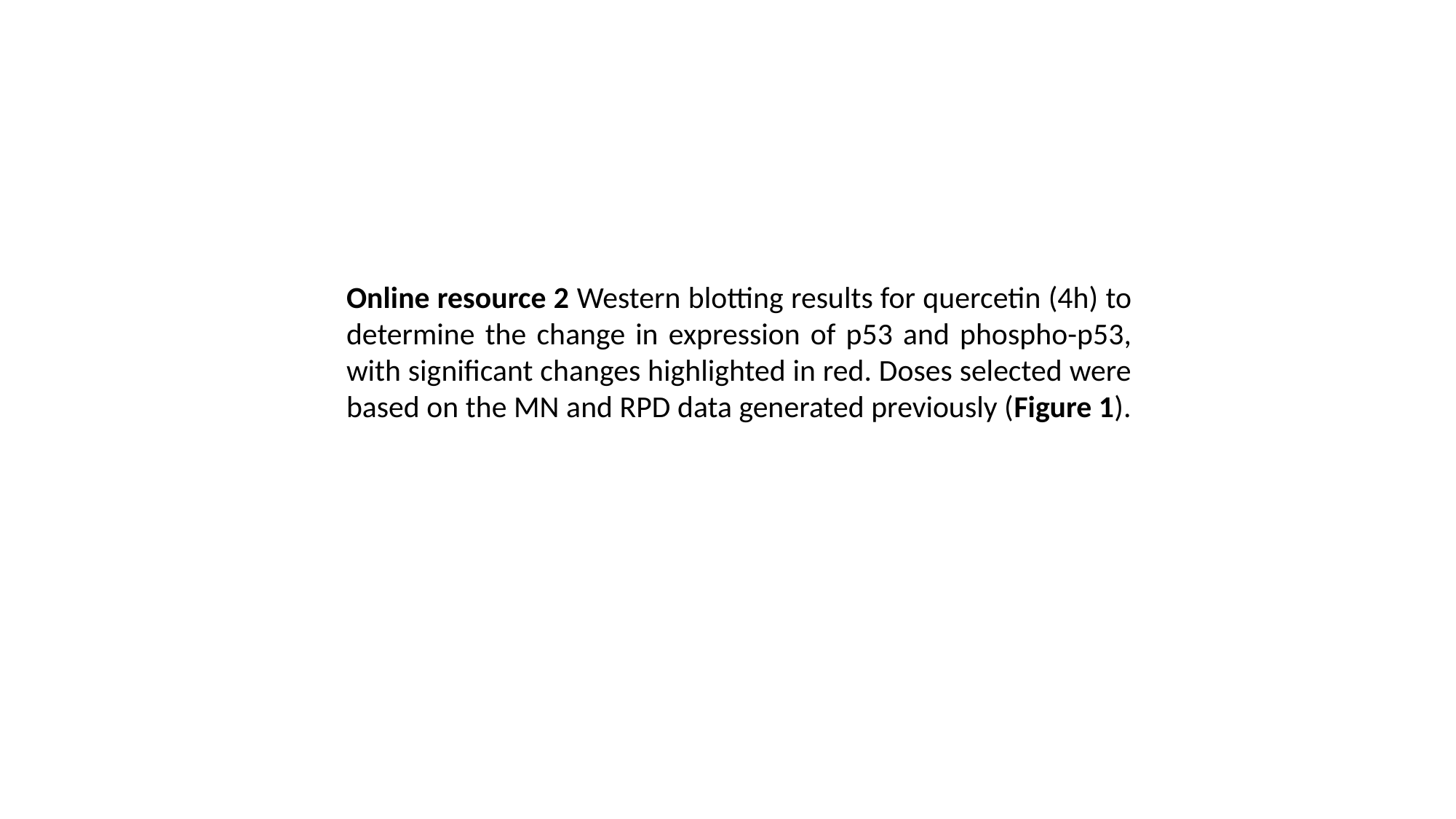

Online resource 2 Western blotting results for quercetin (4h) to determine the change in expression of p53 and phospho-p53, with significant changes highlighted in red. Doses selected were based on the MN and RPD data generated previously (Figure 1).

## Slide 5
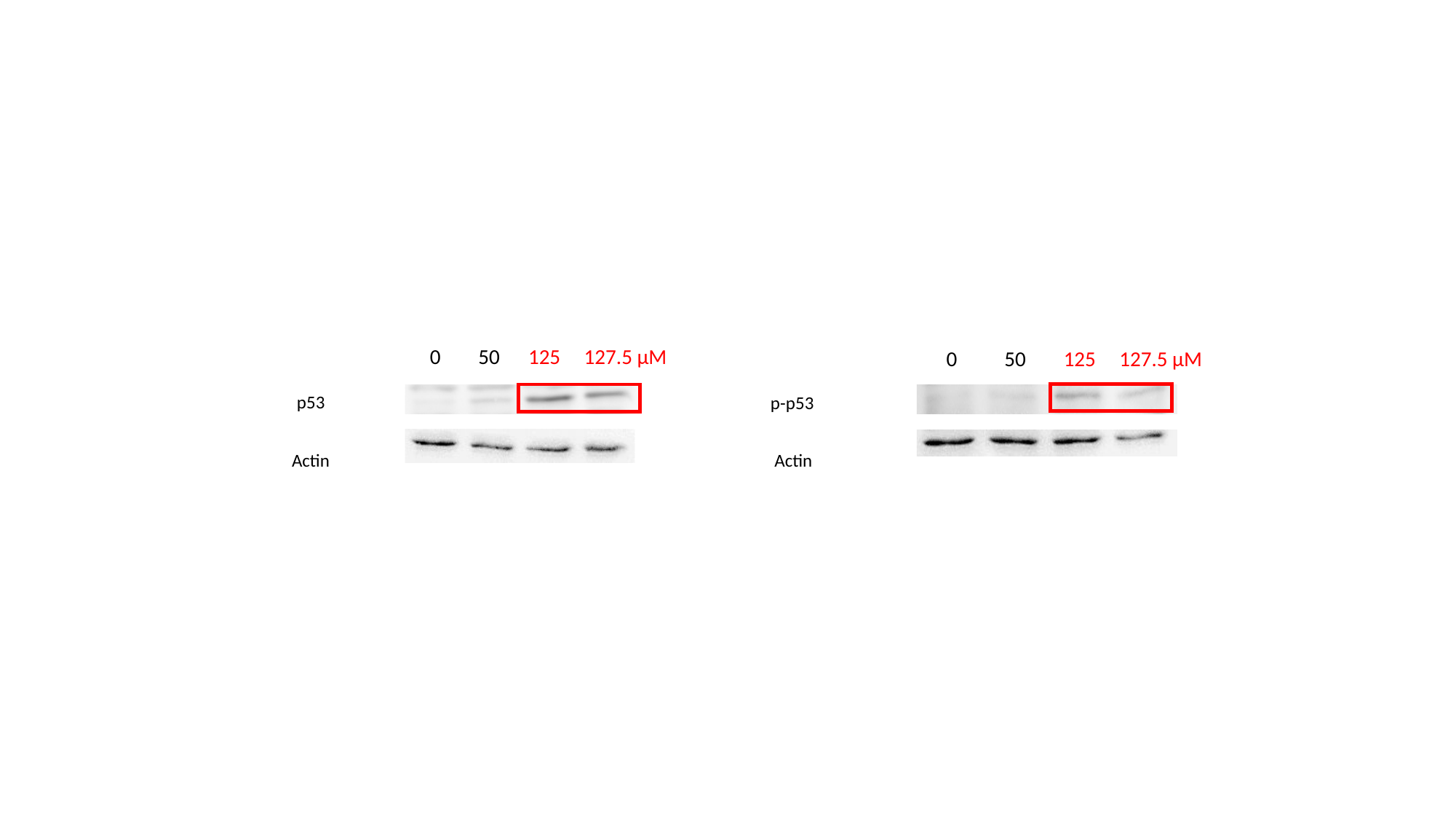

0 50 125 127.5 µM
 0 50 125 127.5 µM
p53
p-p53
Actin
Actin

## Slide 6
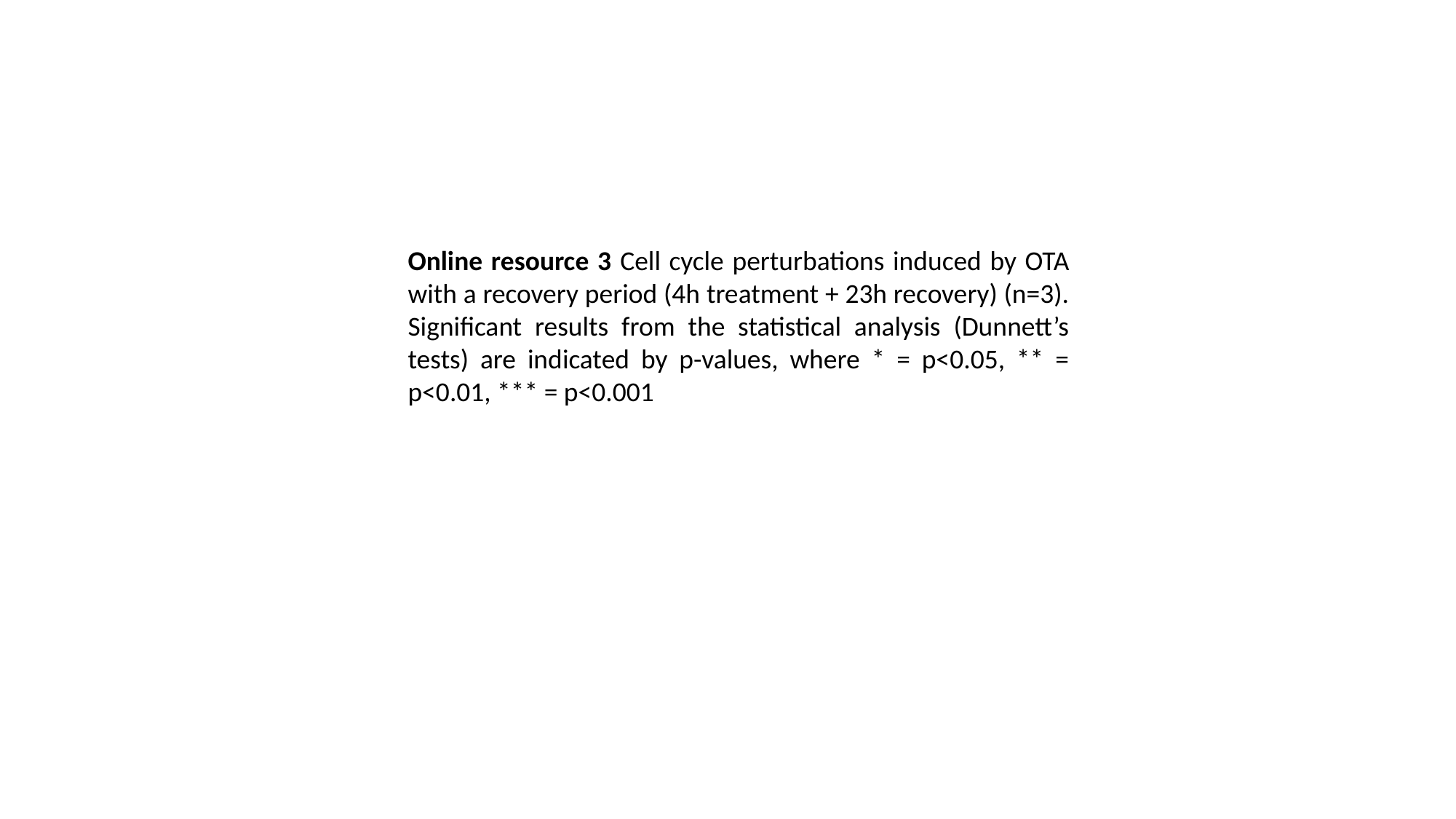

Online resource 3 Cell cycle perturbations induced by OTA with a recovery period (4h treatment + 23h recovery) (n=3). Significant results from the statistical analysis (Dunnett’s tests) are indicated by p-values, where * = p<0.05, ** = p<0.01, *** = p<0.001

## Slide 7
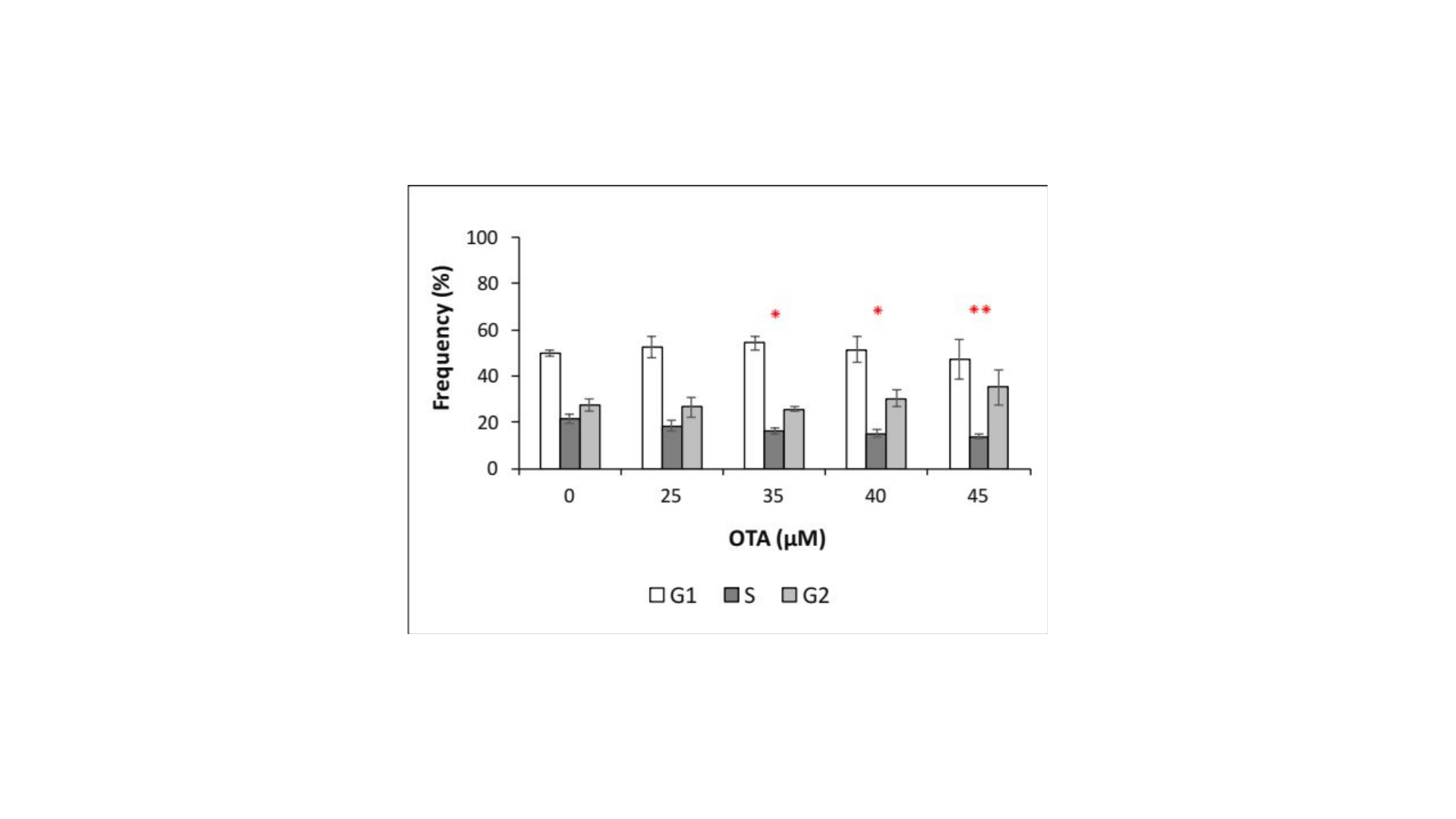

Supplement: Supplementary file 1 — Supplementary file1 (PPTX 138 kb) [file 204_2020_2902_MOESM1_ESM.pptx]
